# Sensitisation to *Blattella germanica* among adults with asthma in Yaounde, Cameroon: a cross-sectional study

**DOI:** 10.1186/1939-4551-7-22

**Published:** 2014-08-19

**Authors:** Eric Walter Pefura-Yone, André Pascal Kengne, Emmanuel Afane-Ze, Christopher Kuaban

**Affiliations:** 1 Department of Internal Medicine and Subspecialties, Faculty of Medicine and Biomedical Sciences, University of Yaounde I, Yaounde, Cameroon; 2 Pneumology service, Yaounde Jamot Hospital, P.O Box: 4021, Yaounde, Cameroon; 3 South African Medical Research Council & University of Cape Town, Cape Town, South Africa; 4 Faculty of health sciences, University of Bamenda, Bamenda, Cameroon

**Keywords:** Asthma, Blattella, Allergy, Aeroallergens, Africa

## Abstract

**Background:**

German cockroach or *Blattella germanica* is commonly found in homes across the inter-tropical region. The contribution of sensitisation to *Blattella germanica* in people with asthma in sub-Saharan Africa has not received attention. The aim of this study was to assess the prevalence and investigate the predicting factors of sensitisation to *Blattella germanica* in patients with asthma in Yaounde, Cameroon.

**Methods:**

This was a cross-sectional study conducted between January 2012 and June 2013. All patients (aged 15 years and above) with asthma, receiving care at the Yaounde Jamot Hospital and the CEDIMER medical practice during the study period and who had received a prick skin testing for perennial aeroallergens were included in the study.

**Results:**

The final sample comprised 184 patients including 123 (66.8%) women. The median age (25th-75th percentiles) was 38 (24–54) years. Prick skin test for *Blattella germanica* was positive in 47 (25.5%) patients. Sensitisation to *Blattella germanica* was associated with a sensitisation to mite in 41 (87.2%) patients, a sensitisation to *Alternaria* in 18 (38.3%) patients, and a sensitisation to cat or dog dander in 7 (14.9%) patients. Independent predicting factors of a sensitisation to *Blattella germanica* were the sensitisation to *Blomia tropicalis* [adjusted odd ratio (95% confidence interval) 4.10 (1.67-10.04), p = 0.002] and sensitisation to *Alternaria* [3.67 (1.53-7.46), p = 0.003].

**Conclusions:**

Sensitisation to *Blattella germanica* is present in about a quarter of adult patients with asthma in Yaounde. Sensitisation to *Alternaria* and *Blomia tropicalis* appears to be a powerful predicting factor of sensitisation to *Blattella germanica* in this setting.

## Introduction

Cockroaches have been identified as one of the major indoor allergens inducing respiratory allergies [1]. Cockroaches are cosmopolitan and are found in large number in warm and humid dwellings, particularly in urban setting [2]. Among the 3,500 known cockroach species, five species (the German cockroach or *Blattella germanica*, the American or *Periplaneta americana*, the Oriental or *Blatta orientalis*, the smokey brown or *Periplaneta fuliginosa*, and the brown-banded or *Supella longipalpis*) are involved in the sensitisation of atopic patients [1-3]. The prevalence of the sensitisation to these species varies across regions worldwide and ranges between 30 to 60% in patients with asthma [2,4,5]. *Blattella germanica* is one of the cockroach species commonly found in homes in Cameroon and in sub-Saharan Africa (SSA) at large [6]. The profile of the sensitisation to *Blattella germanica* in patients with respiratory allergy (asthma or rhinitis) has been largely described in most regions around the world. However, we are not aware of studies on frequency and predicting factors of the sensitisation to *Blattella germanica* in patients with asthma in sub-Saharan Africa at large and in Cameroon in particular. Therefore, the aim of this study was to assess the prevalence and investigate the determinants of sensitisation to *Blattella germanica* in adult asthmatic individuals in Yaounde, Cameroon.

## Material and methods

### Study setting and participants

This study was approved by the ethics committee of the Yaounde Jamot Hospital. It was a cross-sectional study of 18 months duration between January 2012 and June 2013. The study was conducted at the outpatient sections of the Yaounde Jamot Hospital and the private medical practice CEDIMER, both based in Yaounde which is the Capital city of Cameroon. All patients aged 15 years and above, followed for asthma at both institutions during the recruitment period, were invited to take part in the study. Diagnosis of asthma used the Global Initiative for Asthma (GINA) criteria, and was conducted by an experienced chest specialist physician (EWPY). Confirmation of the diagnosis of asthma was based on the GINA criteria [7] and included the presence of episodes of cough, dyspnoea and wheezing associated with reversible airflow obstruction, or non-specific bronchial hyper-responsiveness [7,8]. Patients in whom allergologic skin tests were not conducted were excluded from the study.

### Methods

The following data were collected from all patients: demographic and clinical data including age (in years), sex, ethnicity, duration of asthma, family history of asthma, history of other allergic disease (rhinitis, conjunctivitis, atopic dermatitis) and smoking habits. A diagnosis of rhinitis was based on the following nasal symptoms: colorless rhinorrhoea, nasal congestion, sneezing and itching. The severity of rhinitis was assessed following the classification of the Allergic Rhinitis and its Impact on Asthma (ARIA) group. Rhinitis was considered as persisting if the above symptoms have been present for more than four weeks and for a minimal of four consecutive weeks, otherwise, it was considered as intermittent [9]. The same criteria were applied for intermittent and persisting conjunctivitis and in the presence of symptoms suggesting an ocular allergy including itching, flush, lacrimation and eyelid swelling [10]. The United Kingdom Working Party clinical criteria were applied for the diagnosis of atopic dermatitis [11].

Control of asthma was assessed with the use of the asthma control questionnaire (ACQ) [12,13]. Asthma was considered to be insufficiently controlled during the preceding week in the presence of an ACQ score ≥ 1 [12,13]. The basal (in the absence of any exacerbation) forced expiratory volume in 1 second (FEV1) was recorded for all participants. Basal FEV1 was calculated as the ratio of the measured FEV1 and theoretical FEV1 of each patient. The theoretical FEV1 was estimated with the relevant 2012 Global Lung Initiative equations [14].

Prick tests were performed using the allergenic extracts of Stallergenes Laboratory (Anthony, France). The following perennial aeroallergens were tested: mites (*Dermatophagoïdes pteronyssinus*, *Dermatophagoïdes farinae* and *Blomia tropicalis*), fungi (*Alternaria alternata*), *Blattella germanica*, cat dander and dog dander. A 1% histamine solution was used as positive control, while a phenolic glycerosaline solution was used as negative control. A prick-test was considered positive in the presence of a papule with a diameter greater than 3 mm as compared with that of the negative control, or 50% the diameter of the positive control.

### Statistical analysis

Data analysis used the Statistical Package for Social Sciences (SPSS) v.17 (SPSS Inc., Chicago, USA). Results are presented as median (interquartile range) or count (percentages). The Chi-square test or Fisher exact test were used to compare proportions and the Mann–Whitney U test used to compare quantitative variables. Logistic regressions models were used to investigate the determinants of sensitisation to *Blattella germanica*. Candidate predictors included demographic and clinical variables (sex, age, ethnicity, age at the diagnosis of asthma, family history of asthma, history of rhinitis or atopic dermatitis, smoking, asthma control and basal FEV1), and allergologic variables (sensitisation to mites, sensitisation to Alternaria, sensitisation to animal dander). Associated factors with a p-value <0.10 in univariable analyses were entered altogether in the same multivariable models and the significant one retained as independent associated factors. A p-value <0.05 was used to characterise statistically significant results.

## Results

### General characteristic of the study population

Of the 190 asthmatic adults received in the outpatient departments during the study period, 6 (3.2%) were excluded for missing skin prick testing. Therefore, the final sample comprised 184 patients, of whom 123 (66.8%) were women and 51 (33.2%) were men. Their median age (25th-75th percentiles) was 38 (24–54) years. The median age at the onset of asthma was 22 (12–38) years, and 19 (10.9%) and 63 (39.4%) patients had intermittent and persistent rhinitis respectively. In the week preceding inclusion their enrolment, 97 (57.7%) patients had well-controlled asthma and the median FEV1 was 88.1% (73.5-99.3) (Table 1).

**Table 1:** **Demographic and clinical characteristics of asthmatic adults in Yaounde according to the sensitisation to *Blattella germanica *** **(BG)**

**Characteristics**	**Total sample n = 184 (%)**	**Sensitisation to BG n = 47 (%)**	**No sensitisation to BG n = 137 (%)**	**p**
Sex				
Men	51 (33.2)	15 (31.9)	46 (33.6)	0.835
Women	123 (66.8)	32 (68.81)	91 (66.4)	
Median age, years, (25th-75th percentiles)	38 (24–54)	32 (24–50)	41 (23.5-56)	0.119
Ethnic groups				
Semi-bantou	130 (71.4)	32 (69.6)	98 (72.1)	Reference
Bantou	37 (20.3)	10 (21.7)	27 (19.9)	0.765
Fulani/Sudanese	15 (8.2)	4 (8.7)	11 (8.1)	0.862
Others	2 (1.1)	1 (2.1)	1 (0.7)	0.439
Median age at the onset of asthma, years (25th-75th percentiles)	24 (14–40)	23 (16–39)	24 (13–41.5)	0.720
Rhinitis				
None	93 (50.5)	22 (46.8)	71 (51.8)	Reference
Intermittent rhinitis	25 (13.8)	7 (14.9)	18 (13.1)	0.654
Persistent rhinitis	66 (35.9)	18 (38.3)	48 (35.0)	0.604
Atopic dermatitis				
Yes	4 (2.2)	3 (6.4)	1 (0.7)	0.052
No	180 (97.8)	44 (93.6)	136 (99.3)	
Active smoking				
Non-smoker	157 (98.1)	42 (97.7)	115 (98.3)	Reference
Smoker/ex-smoker	3 (1.6)	1 (2.1)	2 (1.5)	0.612
Second hand smoker	4 (2.2)	2 (4.3)	2 (1.5)	0.301
Family history of asthma				
Yes	33 (17.9)	7 (14.9)	26 (19.0)	0.529
No	151 (82.1)	40 (85.1)	111 (81.0)	
Asthma control				
Well controlled	97 (57.7)	25 (55.6)	72 (58.5)	0.729
Not well controlled	71 (42.3)	20 (44.4)	51 (41.5)	
FEV1, %, median, (25th-75th percentiles)	88.1(73.5-99.3)	88,1 (77–99.5)	88.1 (70.5-99.3)	0.225

FEV1, forced expiratory volume in 1 s.

### Prevalence of sensitisation to Blattella germanica

The prevalence (95% confidence interval) of sensitisation to *Blattella germanica* was 25.5% (19.2-31.8%). Sensitisation to *Blattella germanica* was associated with a sensitisation to mite in 41 (87.2%) patients, a sensitisation to *Alternaria* in 18 (38.3%) patients, and a sensitisation to cat or dog dander in 7 (14.9%) patients.

### Predicting factors of sensitisation to Blattella germanica

There was no difference between patients sensitised and those who were not sensitised to *Blattella germanica* with regard to sex, age, age at clinical onset of asthma and family history of asthma. There was a non-significant high prevalence of history of atopic dermatitis in patients sensitised to *Blattella germanica* compared to those not sensitised (6.4% vs 0.7%, p = 0.052) (Table 1).

Univariable and multivariable associations between sensitisation to *Blattella germanica* and other non-pollinic related aeroallergens are shown in Table 2 and Table 3. The independent predicting factors of sensitisation to *Blattella germanica* were: sensitisation to *Blomia tropicalis* [mutually adjusted odd ratio (95% confidence interval) 4.10 (1.67-10.04), p = 0.002] and sensitisation to *Alternaria* [3.67 (1.53-8.81), p = 0.004].

**Table 2:** **Univariable association between sensitisation to other aeroallergens and sensitisation to *Blattella germanica *** **(BG)**

**Characteristics**	**Total n = 184 (%)**	**Sensitisation to BG n = 47 (%)**	**No sensitisation to BG n = 137 (%)**	**Crude OR (95% CI)**	**p**
Sensitisation to *Dermatophagoïdes pteronyssinus*	93 (50.5)	33 (70.2)	60 (43.8)	3.03 (1.49-6.16)	0.002
Sensitisation to *Dermatophagoïdes farinae*	88 (47.8)	31 (66.0)	57 (41.6)	2.72 (1.36-5.44)	0.004
Sensitisation to *Blomia tropicalis*	85 (46.2)	36 (76.6)	49 (35.8)	5.88 (2.75-12.57)	<0.001
Sensitisation to *Alternaria*	32 (17.4)	18 (38.3)	14 (10.2)	5.43 (2.43-12.22)	<0.001
Sensitisation to cat dander	13 (7.1)	6 (12.8)	7 (5.1)	2.72 (0.86-8.55)	0.099
Sensitisation to dog dander	7 (3.8)	2 (4.3)	5 (3.6)	1.17 (0.22-6.26)	1.000

OR, odds ratio.

**Table 3:** **Multivariable adjusted predicting factors of the sensitisation to *Blattella germanica***

**Predicting factor**	**Adjusted odds ratio (95% confidence interval)**	**p**
Sensitisation to *Dermatophagoïdes pteronyssinus*	1.09 (0.38-3,11)	0.868
Sensitisation to *Dermatophagoïdes farinae*	1.21 (0.44-3.36)	0.715
Sensitisation to *Blomia tropicalis*	4.10 (1.67-10.04)	0.002
Sensitisation to *Alternaria*	3.67 (1.53-8.81)	0.004
Sensitisation to cat dander	2.07 (0.58-7.46)	0.266

## Discussion

The main findings from this study conducted in a developing country of sub-Saharan Africa are the following: 1) about a quarter of asthmatic adults in urban setting are sensitised to *Blattella germanica*; 2) sensitisation to *Blattella germanica* is unrelated to the known age at the onset of asthma; and 3) sensitisation to *Blattella germanica* is associated with a sensitisation to *Blomia tropicalis* and *Alternaria*.

The prevalence of sensitisation to *Blattella germanica* found in our study is comparable to the 23% rate found among asthmatic patients in China [15]. The prevalence of sensitisation to cockroach in studies among atopic patients has been found to be 17.4% in Portugal [16], 20.2% in Turkish [17], 36.8% in the United States [18] and 55% in Brazil [19]. Sensitisation to *Blattella germanica* was not associated with the known age at the onset of asthma in our study. Therefore, the onset of asthma either in the childhood, adolescent or adult age does not seem to affect the sensitisation to *Blattella germanica.* In a study conducted by Sun et al. in the general population in China, sensitisation to cockroach was found to be more frequent among adults than in children [15].

In this study, sensitisation to *Blattella germanica* occurred in the context of multiple sensitisations with sensitisation to *Blomia tropicalis* and Alternaria. Sensitisation to *Blomia tropicalis* was independently associated with sensitisation to *Blattella germanica* in our study. Indeed, 76.6% of patients sensitised to *Blattella germanica* were also sensitised to *Blomia tropicalis* as opposed to only 35.8% for patients not sensitised to *Blattella germanica*. This association is explained at least in part by the cross-reactivity between mite and cockroaches via tropomyosin, but may also reflect true multiple sensitisations [2,19,20]. In the study by Julia-Serda et al., sensitisation to cockroach was also found to be associated with a sensitisation to *Blomia tropicalis*[21].

We also found an association between sensitisation to *Blattella germanica* and sensitisation to *Alternaria*. We found no existing report describing multiple sensitisation linking *Blattella germanica* and *Alternaria*. The two species are very distant phylogenetically, and the association we found appears not to be related to a cross-reaction, but rather to multiple sensitisations in atopic patients. Confirmations of our findings in other settings are needed. Furthermore, employing recombinant major allergens of the two aeroallergens may help to elucidate this association.

The use of prick-test alone to diagnose allergenic sensitisation is a limitation of the current study. Indeed, concomitant measurement of specific immunoglobulin E could have diagnosed other sensitisations among patients with cutaneous hyporeactivity. The two recruitment centres for this study have a primary focus on the management of the adult’s conditions, which precluded the inclusion of children in our study. It is therefore possible that finding from the current study may not be generalizable to the paediatric population with asthma in the same setting. Furthermore, we have not included non-asthmatic participants in the study; therefore, the association between asthma and cockroach atopy could not be assessed in this study.

## Conclusions

In conclusion, sensitisation to *Blattella germanica* is frequent among adult asthmatic patients in Yaounde. This sensitisation appear to be unrelated to the known age of onset of asthma, but rather associated with sensitisation to *Alternaria* and to *Blomia tropicalis*; a finding that requires confirmation and further investigation.

## Consent

Written informed consent was obtained from the patients or dear tutors for the publication of this report and any accompanying images.

## Abbreviations

ACQ: Asthma control questionnaire; FEV1: Forced expiratory volume in 1 second; GINA: Global Initiative for Asthma; SPSS: Statistical Package for Social Sciences; SSA: Sub-Saharan Africa.

## Competing interests

The authors declare that they have no competing interests.

## Authors’ contribution

EWPY conceived the study, collected data, co-analysed the data and drafted of the manuscript; APK contributed to study designed, data analysis, drafting and critical revision of the manuscript; EAZ supervised the data collection and critically revised the manuscript; CK supervised the data collection and critically revised the manuscript. All authors read and approved the final manuscript.
